# Post-dispersal seed removal by ground-feeding rodents in tropical peatlands, Central Kalimantan, Indonesia

**DOI:** 10.1038/srep14152

**Published:** 2015-09-15

**Authors:** Grace V. Blackham, Richard T. Corlett

**Affiliations:** 1 Department of Biological Sciences, National University of Singapore, 14 Science Drive 4, Singapore 117543; 2 Center for Integrative Conservation, Xishuangbanna Tropical Botanical Garden, Chinese Academy of Sciences, Menglun, Yunnan 666303, China

## Abstract

Forested tropical peatlands in Southeast Asia are being rapidly converted to agriculture or degraded into non-forest vegetation. Although large areas have been abandoned, there is little evidence for subsequent forest recovery. As part of a study of forest degradation and recovery, we used seed removal experiments and rodent surveys to investigate the potential role of post-dispersal seed predation in limiting the regeneration of woody plants. Two 14-day seed removal trials were done in deforested and forested peatland habitat in Central Kalimantan, Indonesia. Seeds of *Nephelium lappaceum*, *Syzygium muelleri*, *Artocarpus heterophyllus* (all animal-dispersed) and *Combretocarpus rotundatus* (wind-dispersed) were tested. Significantly more seeds (82.8%) were removed in forest than non-forest (38.1%) and *Combretocarpus* had the lowest removal in both habitats. Most handled seeds were eaten *in situ* and little caching was observed. Six species of rodents were captured in forest and five in non-forest. The most trapped taxa were three *Maxomys* spp. in forest (85.5% of individuals) and *Rattus tiomanicus* in non-forest (74.8%). Camera traps confirmed that rodents were responsible for seed removal. Seed predation in deforested areas, which have a much lower seed rain than forest, may contribute to the low density and diversity of regenerating forest.

Tropical peatlands cover around 250,000 km^2^ of SE Asia, but have been severely impacted over the last few decades by logging and conversion to agriculture^1^,^2^. Agricultural conversion of deep peat is difficult, however, and vast areas have been abandoned after clearance. At least 18,000 km^2^ of the 160,000 km^2^ of peatlands found in Peninsula Malaysia, and on the islands of Borneo and Sumatra, has now been severely degraded^3^, particularly in Riau and Jambi in Sumatra and in Central, East and South Kalimantan in Indonesian Borneo. Moreover remote sensing provides little evidence for extensive forest recovery after abandonment^3^.

Previous studies of natural regeneration in degraded tropical peatlands have shown that ferns, climbers, sedges and grasses dominate after disturbance, and that these areas support fewer woody plant species than intact peat swamp forest ecosystems^4^,^5^. The seed rain into an extensive degraded area in Kalimantan was species-poor, with three-quarters of the seeds from only two wind-dispersed species^6^. This was attributed to a combination of isolation from forest seed sources and the dominance of the regrowth avifauna by bulbuls and other small passerines, while large-gaped birds and mammalian frugivores were absent^7^. Woody regrowth was also dominated by a few abundant wind-dispersed species, while most other species were potentially dispersed by bulbuls, but additional woody species had apparently sprouted from vegetative remnants of the previous forest cover^8^.

The contrast between the densities of the seed rain (mean 95 seeds m^−2^ yr^−1^ in the open and 1128 seeds m^−2^ yr^−1^ under trees) and woody regrowth (mean 0.09 individuals m^−2^, representing several years seed rain) in non-forest habitats in these studies^6^,^8^ suggests that post-dispersal seed predation may be a significant factor limiting forest recovery. Several tropical studies have suggested that seed predation is a filter limiting natural regeneration of woody plants in degraded areas^9^,^10^. Post-dispersal seed predators in SE Asia include rodents, pigs, ants and other insects, but rodents appear to be the dominant predators of seeds in the size range of the woody seed rain in degraded sites^11^.

This study therefore used seed removal experiments and rodent surveys to investigate post-dispersal seed removal in the same tropical peatland habitat in Central Kalimantan, Indonesia, where the seed rain and woody regeneration were previously studied^6^,^8^. Specifically, it aimed to: (1) determine whether levels of post-dispersal seed removal differed between forested and deforested habitats; (2) establish the species composition of the ground-feeding rodents in both habitats; (3) establish whether removal was secondary seed dispersal or predation; (4) assess the possible implications for forest recovery.

## Methods

### Study area

This study was carried out in the former Mega Rice Project (MRP) area in Central Kalimantan, Indonesia. The MRP was initiated in 1996 and aimed to develop one million hectares of peat swamp forest into land for rice cultivation. This involved widespread forest clearance and the excavation of canals to drain the naturally water-logged peat^12^. The project was cancelled in 1999, but by this time most of the area had already been logged and cleared^13^. Two habitat types were used in this study: (1) logged peat swamp forest (hereafter ‘forest’) and (2) non-forest regrowth (‘non-forest’), following other studies at this site^6^,^7^,^8^.

The forest was located in the Tuanan Study Area, part of the 300,000 ha Mawas Conservation Area in the former MRP (02°09′S; 114°26′E). This forest was not cleared during establishment of the MRP, but was selectively-logged in the 1990s and illegally logged subsequently^14^. All logging stopped in 2002^15^. The non-forest was in Block A North-West (2°17′S; 114°31′E) of the former MRP. Tuanan and Block A NW are both located on the Mantangai peat dome, although they are now separated by the two primary canals. Block A NW covers approximately 45,000 ha and was subjected to forest clearance and widespread drainage. An elaborate grid system of 300 km of canals divides the area into compartments roughly 2.5 by 2.5 km^16^. The non-forest habitat was dominated by ferns, with smaller areas occupied by woody plants, bare ground, and standing or fallen dead wood^8^.

### Seed removal trials

Two pre-existing transects in the forest and two purposely cut transects in the non-forest were used in this study. Each transect was used for a single seed removal experimental trial lasting 14 days. The four trials took place between May and November 2011 and were not run concurrently due to logistical issues. All trials included seeds of one native animal-dispersed peat swamp species fruiting at the time of study, *Nephelium lappaceum* or *Syzygium muelleri*, seeds of *Combretocarpus rotundatus* (wind-dispersed), which is the dominant woody plant species in non-forest, and seeds of locally bought *Artocarpus heterophyllus* (jackfruit). Mean seed sizes are shown in Table 1. *Artocarpus* has been used in other studies on seed predation^17^,^18^ since it seems to be a reasonable model for large seeds and was chosen because seeds of native species were not always available. Fruits of the native species were collected by climbing trees to retrieve ripe fruits. Seeds were removed from fruit and cleaned to remove all fleshy parts, except for *Combretocarpus*, which is a winged seed with no flesh. One metre of cotton thread ending in numbered flagging tape was attached to each seed with non-toxic glue so removed seeds could be tracked. Other seed removal studies have found no difference between the removal of marked and unmarked seeds^19^,^20^.

A set of nine seeds made up of three of each species (always *Combretocarpus* and *Artocarpus*, with the third either *Nephelium* or *Syzygium*) was laid out in a grid, with 50 cm between seeds. Each grid will be referred to as a seed station. Twenty seed stations were laid out at 50 m intervals along each transect, giving a total of 360 seeds (9 × 20 × 2) in each habitat type. Seed stations were at least 1 m from the main transect line. Where necessary, a 2 m^2^ area was cleared of above-ground vegetation to provide a substrate to place seeds on. Leaf litter and over-hanging vegetation were left intact.

Seed stations were checked daily for 14 days, with seeds classed as handled or not. Handled seeds were further classed as eaten at the station or removed. Where seeds were removed, the plan was to search up to 20 m radius, on the assumption that rodents rarely carry seeds further^18^, but in practice all were found within 5.5 m. Removed seeds were further classed as eaten if seed fragments were found and cached if seeds were found below the soil surface or below leaf litter. Cached seeds were checked for the remainder of the study. Seeds with evidence of seed coat damage (by insects or rodents) were also classed as preyed upon^9^ and not counted as surviving, except in *Combretocarpus* where damage to the wings was not counted as long as the seed remained intact.

Ten camera traps (Bushnell Trophy camera, 5 megapixel model, Bushnell, Inc.) were installed along one seed removal experimental trial transect in forest and one in non-forest. A camera trap was placed at every second seed station, 10–20 cm from the ground and with a view of the seeds. Cameras were set to take a 20 second video when the sensor was triggered.

### Rodent surveys

Trapping was done along each transect to assess the composition of the ground-feeding rodent community. Surveys were carried out within a month of the seed removal trial on each transect, using Tomahawk collapsible live traps model 201 (Tomahawk Live Traps IIC) covered with locally available materials (fallen leaves, ferns, dead wood etc.) for camouflage and rain protection. On each transect, 90 traps were set at 10 m intervals on the ground, at least 2 m from the transect line. A seven day session (630 trap nights) was conducted along each transect. Traps were baited with a mixture of banana and peanut butter, opened at 16.30 and checked the following morning at 07.00. Animals were marked with Monel ear tags (National Band and Tag Co., USA), measured and then released. Species were identified using *A Field Guide to the Mammals of Borneo*^21^ with body, tail, ear and foot measurements taken. All captured animals were also photographed.

### Data analysis

To compare the levels of post-dispersal seed removal in forest and non-forest, data were analysed as the proportion of seeds remaining at each seed station, because the survival of individual seeds at each seed station could not be considered as independent of one another. Data were pooled for each habitat, giving 40 stations for forest and 40 for non-forest. A Mann-Whitney U test was used to compare the numbers of seeds remaining with an intact seed coat after 14 days in forest and non-forest, with stations as replicates (SPSS. V16). To look at variation in levels of post-dispersal seed loss amongst species the cumulative percentage of seeds handled (i.e. eaten at the station or removed from it) over time was calculated for each species and habitat. To establish the overall fate of seeds in the removal trials, percentages of each seed fate were calculated for each habitat.

## Results

### Seed fates

**Figure 1:**
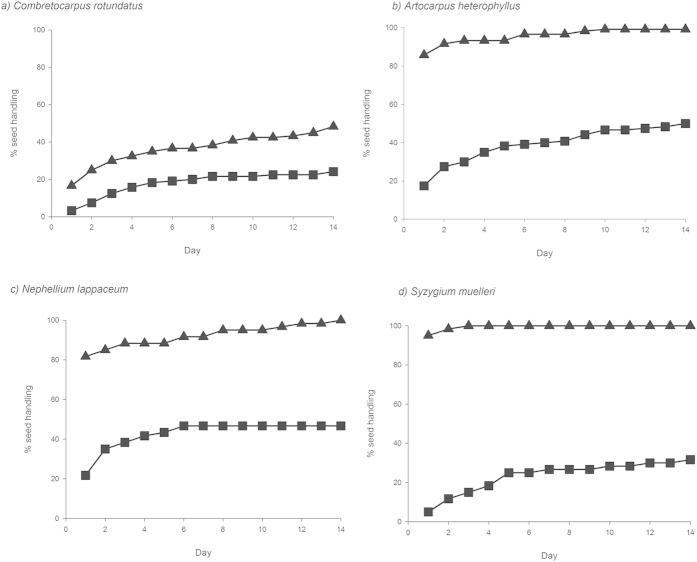
Cumulative percentage of seeds handled (i.e. eaten or removed) over time for (**a**) *Combretocarpus rotundatus*, (**b**) *Artocarpus heterophyllus*, (**c**) *Nephelium lappaceum* and (d) *Syzygium muelleri*. ▲ = forest, ■ = non-forest.

We were able to locate all tags (within 5.5 m of the seed station) and thus to establish the fates of all the seeds. Significantly more seeds (82.8%) were handled (i.e. eaten or removed) in forest than non-forest (38.1%) after 14 days (Mann Whitney U, P < 0.05) (Table 2). The mean number of seeds remaining with an intact seed coat at a seed station (out of a possible 9) was 1.6 (±1.1) in forest and 5.6 (±2.8) in non-forest. *Combretocarpus* had the highest proportion of seeds remaining at seed stations in both habitats, with more than half the seeds (51.7%) remaining in forest and more than three-quarters (75.8%) in non-forest. In contrast, no seeds of *Nephelium* and *Syzygium* and only one of *Artocarpus* (0.8%) remained in forest, and 53.3%, 68.3%, and 50.0% remained in non-forest (Fig. 1). In forest more seeds were handled than not handled, while in non-forest more seeds were not handled than handled. In both forest and non-forest most handled seeds were eaten at the seed station (191 and 118 respectively). More seeds were eaten away from the seed station in forest (99, 33% of handled seeds) than in non-forest (17, 12% of handled seeds) and more seeds were cached in forest (8, 0.027%) than non-forest (2, 0.015%). Of the cached seeds, only a single *Artocarpus* in forest remained cached and intact at the end of the study, 0.5 m from its original location.

Videos obtained by camera traps showed four instances of small mammals, consistent with the commonly trapped *Rattus tiomanicus* from their size, consuming seeds in the non-forest site, and 12 instances in forest, one by a treeshrew and the others by rats, consistent with *Maxomys* spp., from their size and tail coloration. In forest there were also three instances of rats, consistent with *Maxomys* spp., removing seeds without consuming them.

### Rodent surveys

A total of 354 different individual rodents of eight species were captured over 2520 trap-nights (Table 3). All were murids except for four *Callosciurus notatus* tree squirrels in the non-forest site. More individuals and more species were captured in forest (6 species, 205 individuals) than in non-forest (5 species, 147 individuals), although given the short timescale of the study and the large differences in the habitats, these counts should not be treated as estimates of relative abundance or diversity. The most trapped taxa were three *Maxomys* spp. in forest (85.5% of individuals) and *Rattus tiomanicus* in non-forest (74.8%). Two species of treeshrews (*Tupaia* spp.) were also trapped.

## Discussion

The significantly higher level of post-dispersal seed removal in forest than non-forest is comparable with the results of other studies^22^,^23^,^24^. The rate of seed removal found for forest in this study was higher than that for lowland dipterocarp forests in SE Asia^25^,^26^, but not unusual for seed removal experiments in other parts of the tropics^9^,^27^. In contrast, the rate of seed removal in non-forest was lower than degraded landscapes in other parts of the tropics^9^,^23^,^28^. The animal-dispersed seeds, *Artocarpus*, *Syzygium* and *Nephelium*, all had very high levels of handling in forest (99–100%), but lower levels in non-forest (30–50%), while the wind-dispersed *Combretocarpus* had the lowest levels in both habitats. Low rates of seed handling have been reported for wind-dispersed species in other studies^29^.

The two habitats shared five ground-feeding rodent species, but with very different capture rates. Forest-specialist *Maxomys* spp. dominated the forest while the non-forest dominant, *Rattus tiomanicus*, was not recorded in forest. *R. tiomanicus* is typically found in scrubland, grassland, and plantations but rarely in undisturbed forest^21^,^30^. The lack of trees explains the absence of arboreal rats (*Lenothrix canus* and *Niviventer cremoriventer*) in the degraded site. In other forest types in SE Asia, disturbance has caused a loss of native species and the invasion of generalists^30^,^31^, as happened in our sites, or the loss of rare species and the perseverance of common species^32^.

The camera trap videos showed that rodents were responsible for seed removal in both habitats, probably *Maxomys* spp. in forest and *R. tiomanicus* in non-forest. Other studies in SE Asia have also attributed seed removal to rodents. Video cameras were used to record seed removal agents in peat swamp forest in Sarawak, Malaysia, and identified *M. whiteheadi, Sundamys muelleri* and *Callosciurus notatus* as the primary removal agents^18^, all of which were also captured in this study. While the limited use of camera traps did not record seed removal by all species captured, evidence from other studies shows they all consume some seeds^18^,^26^.

In both habitats only a tiny proportion of seeds was cached initially, and only a single seed of *Artocarpus* in forest remained cached and intact by the end of the study. *Rattus* species are not known to scatter-hoard^11^ so the dominance of *R. tiomanicus* in non-forest may explain the absence of caching there. *Maxomys* species scatter-hoard elsewhere^11^, but very little caching of jackfruit seeds was reported in a peat swamp forest in Sarawak, Malaysia^18^. Tropical peatlands are typically water-logged and degraded areas often experience flooding, so the high water-table may make caching impractical. At these sites, therefore, seeds handled by rodents are almost all predated. Rodents swallow and disperse some very small seeds in their faeces^11^, and may contribute to the dispersal of small-seeded species, such as *Melastoma malabathricum* in the non-forest site, but this and other small-seeded species are also dispersed by the abundant bulbuls^7^.

While non-forest had a significantly lower level of seed predation than forest, non-forest sites had much lower seed rain densities, except under trees, and three-quarters of the seeds were from two wind-dispersed species^6^. If the levels of predation of animal-dispersed seeds observed in non-forest areas in this study are typical, the impact on woody regrowth could be large: perhaps enough to contribute significantly to the low density and diversity of the regenerating forest. Future studies should use rodent-exclusion experiments to assess this further.

## Additional Information

**How to cite this article**: Blackham, G. V. and Corlett, R. T. Post-dispersal seed removal by ground-feeding rodents in tropical peatlands, Central Kalimantan, Indonesia. *Sci. Rep.* **5**, 14152; doi: 10.1038/srep14152 (2015).

## Figures and Tables

**Table 1:** Mean seed sizes (with standard deviation) in mm.

**Species**	**Length**	**Width**	**Breadth**
*Combretocarpus rotundatus* (including wings) (20)	20 ± 2.1	25.9 ± 3.6	0.04 ± 0.02
*Nephelium lappaceum* (6)	16.7 ± 2.7	10.0 ± 2.1	10.5 ± 1.9
*Syzygium muelleri* (10)	11.6 ± 1.8	11.8 ± 0.4	11.8 ± 0.6
*Artocarpus heterophyllus* (10)	29.2 ± 1.4	20.8 ± 2.8	13 ± 3.9

Number of seeds measured in parentheses.

**Table 2:** Fates of thread-marked seeds in two tropical peatland habitats, Central Kalimantan, Indonesia.

		**Seed fate**					
		**Not handled**	**Handled**				**Total**
			**Not removed**	**Removed**			
				**Not cached**	**Cached**		
			**Eaten at seed station**	**Eaten away from seed station**	**Eaten by end of study**	**Not eaten by end of study**	
Forest	Total count	62	191	99	7	1	360
	% of total	17.2	53.1	27.5	1.9	0.3	100
Non-forest	Total count	223	118	17	2	0	360
	% of total	61.9	32.8	4.7	0.6	0.0	100

All seeds were re-located.

**Table 3:** Number of individuals of small mammal species trapped.

**Species**		**Total**	**Forest**		**Non-forest**	
			**F1**	**F2**	**NF1**	**NF2**
Muridae						
*Lenothrix canus*	Grey tree rat	2	2	0	0	0
*Maxomys rajah*	Brown spiny rat	83	36	30	7	10
*M. surifer*	Red spiny rat	14	4	9	1	0
*M. whiteheadi*	Whitehead’s rat	113	32	66	2	13
*Niviventer cremoriventer*	Dark-tailed tree rat	22	12	10	0	0
*Rattus tiomanicus*	Malaysian field rat	110	0	0	40	70
*Sundamys muelleri*	Muller’s rat	6	4	2	0	0
Sciuridae						
*Callosciurus notatus*	Plantain squirrel	4	0	0	4	0
Tupaiidae						
*Tupaia minor*	Lesser treeshrew	6	1	4	0	1
*T. splendidula*	Ruddy treeshrew	3	0	2	0	1
Total individuals		363	91	123	54	95
Total species		10	7	7	5	5

There were 630 trap-nights in each of the four transects.
